# Comparative Biocontrol Efficacy and Mechanisms of Indirect and Direct Application Methods Against Leaf Spot Caused by *Pseudomonas syringae* pv. *aptata* in Sugar Beet

**DOI:** 10.3390/ijms27114672

**Published:** 2026-05-22

**Authors:** Tamara Krstić Tomić, Marija Nedeljković, Aleksandra Mesaroš, Jovana Todorović, Marijana Pešaković, Slaviša Stanković, Jelena Lozo

**Affiliations:** 1 Fruit Research Institute, 32000 Čačak, Serbia; tkrstictomic@institut-cacak.org (T.K.T.); jtodorovic@institut-cacak.org (J.T.); mpesakovic@institut-cacak.org (M.P.); 2 University of Belgrade - Faculty of Biology, 11000 Belgrade, Serbia; marija.nedeljkovic@bio.bg.ac.rs (M.N.); aleksandra.mesaros@bio.bg.ac.rs (A.M.); slavisas@bio.bg.ac.rs (S.S.); 3 Center for Pathogen Biocontrol and Plant Growth Promotion, University of Belgrade - Faculty of Biology, 11000 Belgrade, Serbia

**Keywords:** rhizobacteria, seed priming, induced systemic resistance

## Abstract

Using beneficial bacteria from the plant microbiome to combat pathogens is an environmentally friendly strategy for biological control. Although significant progress has been made in characterizing microorganisms with biocontrol potential, the optimal methods for applying such biological preparations to achieve maximum effectiveness against plant pathogens remain insufficiently defined. Our goal was to select rhizobacteria from the sugar beet microbiome and analyze their biocontrol capacity in both indirect and direct applications to protect the plant from *Pseudomonas syringae* pv. *aptata* P21. The methodological approach differed: indirect application involved seed priming with selected strains, *Bacillus safensis* MRh275, *B. pseudomycoides* JRh226, *Stenotrophomonas maltophilia* JRh266, or the T2 consortium (MRh275 and JRh266), while direct application involved simultaneous treatment of both the pathogen and the biocontrol strain. Although the direct approach resulted in a greater reduction in lesions and a lower concentration of H_2_O_2_, the indirect approach showed higher activity of peroxidase and superoxide dismutase as antioxidant enzymes, as well as phenylalanine ammonia-lyase, which is involved in the phenylpropanoid pathway and plant defense mechanisms. Infected plants showed higher expression of *NPR1*, *MYC2*, and *LOX* defense-related genes only under indirect biocontrol with all three strains, except in the T2 application. The T2 consortium performed best in direct biocontrol, where it most effectively reduced lesions. Since encounters between plants and pathogens cannot be accurately predicted, and the application of biological preparations should be easy and accessible for farmers, this study highlights the use of indirect biocontrol through seed priming to enhance the plant’s intrinsic defense capacity.

## 1. Introduction

Among bacterial plant pathogens, *Pseudomonas syringae* is one of the most widespread and economically important species, capable of infecting more than 500 plant species and causing significant agricultural losses [1,2]. Its efficient dissemination through water, rainfall, and aerosols further complicates disease management [3]. In sugar beet, *P. syringae* pv. *aptata* is associated with bacterial leaf spot, a disease that negatively affects crop performance and quality [4]. Because conventional disease control strategies are limited, biological control has emerged as a promising and environmentally sustainable alternative [5,6]. Biocontrol mechanisms are generally categorized as direct, involving antagonistic interactions such as antibiosis and competition with the pathogen, or indirect, based on the activation of plant defense responses [7,8]. Although both approaches have been widely studied, their relative efficacy and underlying mechanisms remain insufficiently understood, particularly within the same experimental framework.

Plant–microorganism interactions are mediated by complex chemical signaling, primarily driven by root exudates containing sugars, amino acids, and secondary metabolites, which shape microbial recruitment and community assembly in the rhizosphere [9]. These processes determine microbial colonization of the rhizosphere, a critical interface where beneficial microorganisms establish, persist, and influence host physiology [10]. After successful colonization, beneficial microbes affect plant health by competing with pathogens for ecological niches and by modulating plant immune responses, forming the basis of indirect biocontrol [11,12]. Although *P. syringae* pv. *aptata* primarily infects aerial tissues, rhizosphere-mediated interactions may indirectly modulate disease development through systemic defense signaling pathways [13].

Despite the increasing use of plant-associated beneficial bacteria, their performance under field conditions remains inconsistent and is strongly dependent on both strain and conditions [14]. This inconsistency suggests that the outcome of biocontrol depends not only on microbial traits but also on the mode of application and the extent to which plant defense mechanisms are involved [15,16]. However, comparative studies that directly distinguish between pathogen suppression and host-mediated resistance, while integrating biochemical and molecular responses, remain limited.

This study evaluated and compared the efficacy and underlying mechanisms of direct and indirect biocontrol strategies against *P. syringae* pv. *aptata* in sugar beet. We hypothesized that indirect biocontrol, through activation and priming of plant defense responses, would provide more consistent and less environmentally dependent protection than direct antagonistic interactions. To test this hypothesis, we analyzed disease development, antioxidant enzyme activity, hydrogen peroxide concentration, and defense-related gene expression. This approach enables the separation of pathogen-targeted effects from plant-mediated resistance mechanisms and provides insight into the relative contributions of pathogen suppression and intrinsic plant defenses, advancing the rational application of biocontrol in crop protection.

## 2. Results

### 2.1. Characterization of Plant Growth-Promoting Traits of Selected Bacterial Strains

Using various cultivation methods and media in a previous study, 403 isolates were obtained from the rhizosphere and 128 from the phyllosphere of sugar beet during one growing season [17]. The strains were characterized for multiple plant growth-promoting (PGP) traits, and 98 strains were selected for analysis of indole-3-acetic acid (IAA) production, nitrogen fixation, ACC deaminase activity, motility, and biofilm formation. These traits, along with previously described antibacterial activity against different *P. syringae* [17], served as criteria for selecting three rhizosphere bacteria, *Bacillus safensis* MRh275, *B. pseudomycoides* JRh226, and *Stenotrophomonas maltophilia* JRh266 for further biocontrol experiments (Table 1). In addition to examining the individual effect, we also investigated the effect of a consortium composed of these strains. We first performed cross-antagonistic and cocultures tests to monitor their joint growth. The results showed that strain *B. pseudomycoides* 226 inhibited the growth of the other two strains (Table S1), so we selected strains *B. safensis* MRh275 and *S. maltophilia* JRh266 for the consortium, which grew successfully in cocultures (Figure S1).

### 2.2. Effect of Individual Strains and a Consortium on Lesion Development in Indirect and Direct Biocontrol

To evaluate the biocontrol potential of individual strains, *B. safensis* MRh275, *B. pseudomycoides* JRh226, and *S. maltophilia* JRh266, as well as the T2 consortium composed of strains MRh275 and JRh266, we conducted experiments using both indirect and direct application approaches.

**Figure 1:**
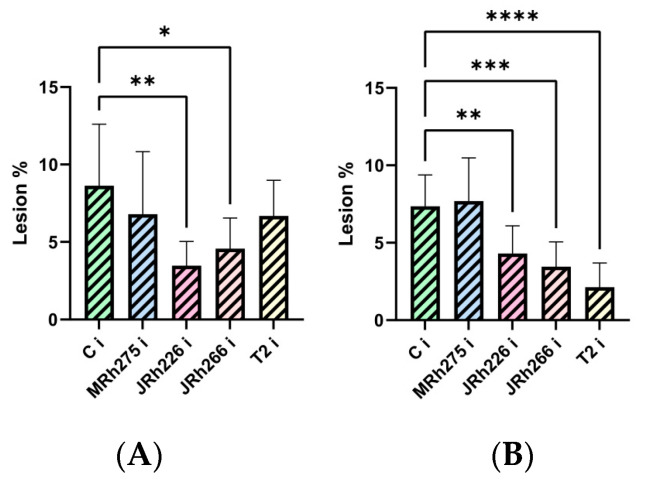
Effect of individual strains and the T2 consortium on lesion development in sugar beet in indirect and direct biocontrol. Indirect biocontrol—all treatments (**A**); direct biocontrol—all treatments (**B**). Significant differences are indicated as follows: *p* < 0.05 (*), *p* < 0.01 (**), *p* < 0.001 (***), *p* < 0.0001 (****).

For indirect biocontrol, sugar beet seeds were soaked in suspensions of individual strains or the consortium, allowing the bacteria to interact with the seeds. After plant development, leaves were inoculated with the pathogen *P. syringae* pv. *aptata* P21, and lesion development was assessed. Compared with the untreated infected control (C i), seeds treated with strains JRh226 and JRh266 showed a statistically significant reduction in lesion size (Figure 1A). For direct biocontrol, the pathogen and the biocontrol strains or consortium were co-inoculated into leaves simultaneously. In this setup, strains JRh226, JRh266, and the T2 consortium significantly reduced lesion size compared with the untreated infected control (C i) (Figure 1B).

### 2.3. Plant Response to Biocontrol Treatments Through Hydrogen Peroxide (H_2_O_2_) Concentration

**Figure 2:**
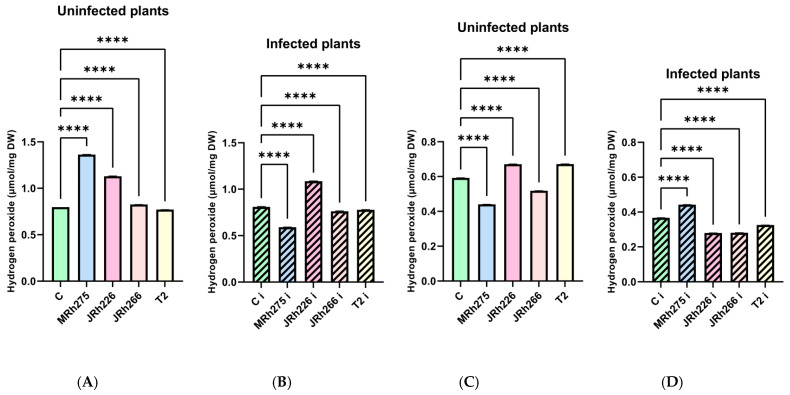
Hydrogen peroxide (H_2_O_2_) accumulation in indirect and direct biocontrol. Different biocontrol isolates influenced H_2_O_2_ levels under both indirect (**A**,**B**) and direct (**C**,**D**) biocontrol conditions. Treatments were compared to the untreated control in uninfected plants (**A**,**C**) and to the infected control (C i) in plants challenged with *P. syringae* pv. *aptata* P21 (**B**,**D**). Significant differences are indicated as follows: *p* < 0.0001 (****).

In the indirect treatment, H_2_O_2_ accumulation is shown in Figure 2. In uninfected plants, levels were significantly higher in treatments with strains MRh275, JRh226, and JRh266 than in the uninfected control, whereas the T2 consortium showed significantly lower values (Figure 2A). Infected plants treated with MRh275, JRh266, and the T2 consortium had significantly lower H_2_O_2_ concentration (Figure 2B), whereas strain JRh226 induced significantly higher accumulation. In plants subjected to direct biocontrol treatment, H_2_O_2_ levels in uninfected plants varied among treatments. Strains MRh275 and JRh266 showed significantly lower levels compared with the uninfected control, while strain JRh226 and the T2 consortium exhibited significantly higher accumulation (Figure 2C). A different pattern was observed in pathogen-infected plants (Figure 2D), where strain MRh275 induced significantly higher H_2_O_2_ levels than the infected control, while other treatments showed significantly lower values.

### 2.4. Effect of Direct and Indirect Biocontrol Treatments on Antioxidant Enzyme Activities

**Figure 3:**
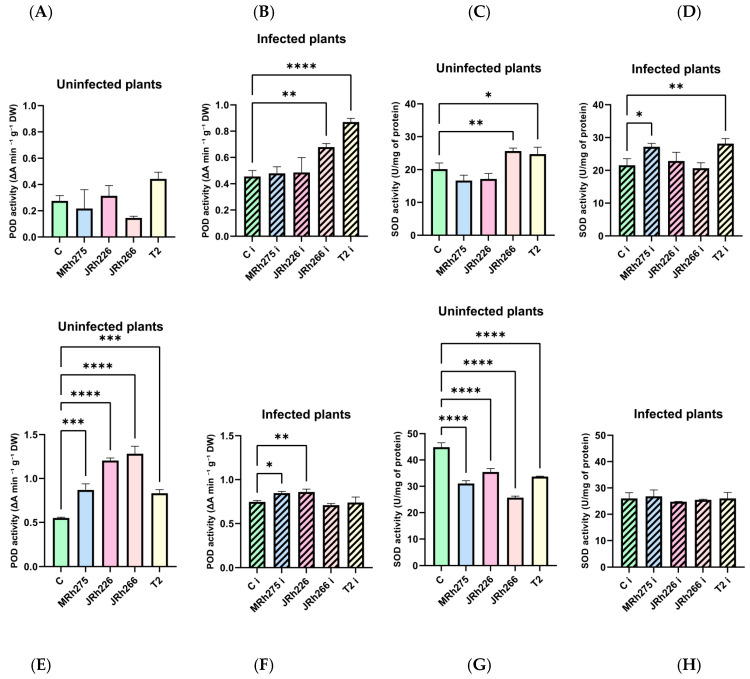
Antioxidative enzyme activities in indirect and direct biocontrol. Plants treated with different biocontrol isolates showed variations in peroxidase and superoxide dismutase activity under both indirect (**A**–**D**) and direct (**E**–**H**) biocontrol approaches. Treatments were compared to the untreated control in uninfected plants (**A**,**C**,**E**,**G**) and to the infected control (C i) in plants challenged with *P. syringae* pv. *aptata* P21 (**B**,**D**,**F**,**H**). Significant differences are indicated as follows: *p* < 0.05 (*), *p* < 0.01 (**), *p* < 0.001 (***), *p* < 0.0001 (****).

The effects of biocontrol treatments on antioxidant enzyme activities were evaluated by measuring peroxidase (POD) and superoxide dismutase (SOD) in both uninfected and pathogen-infected plants. Under the indirect biocontrol approach, POD activity in uninfected treated plants did not differ significantly from that of the uninfected control (Figure 3A). In contrast, SOD activity was enhanced in plants treated with strain JRh266 and the T2 consortium (Figure 3C). Following pathogen infection, both enzymes responded to the treatments, with POD activity significantly elevated in plants pretreated with JRh266 and the T2 consortium (Figure 3B). SOD activity increased in plants treated with strain MRh275 and the T2 consortium (Figure 3D).

Under the direct biocontrol approach, uninfected plants exhibited elevated POD activity across all treatments (Figure 3E), whereas SOD activity was consistently lower than in the uninfected control (Figure 3G). In infected plants, POD was significantly higher in those treated with strains MRh275 or JRh226 (Figure 3F), while SOD showed no significant variation compared to the infected control (Figure 3H).

### 2.5. Phenylalanine Ammonia-Lyase (PAL) Activity Under Different Biocontrol Treatments

**Figure 4:**
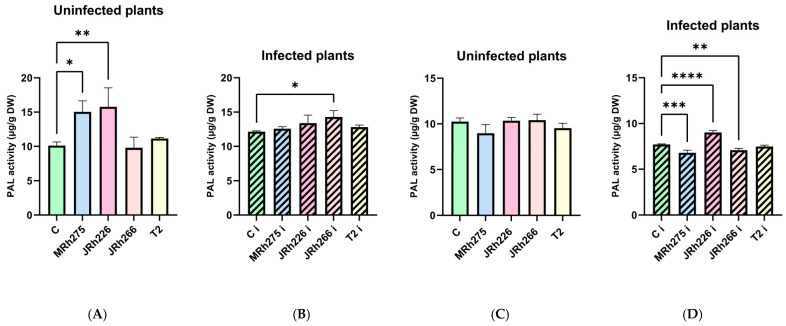
Phenylalanine ammonia-lyase (PAL) activity in indirect and direct biocontrol. Plants treated with different biocontrol isolates showed variations in PAL activity under both indirect (**A**,**B**) and direct (**C**,**D**) biocontrol conditions. Treatments were compared to the untreated control in uninfected plants (**A**,**C**) and to the infected control (C i) in plants challenged with *P. syringae* pv. *aptata* P21 (B, D). Significant differences are indicated as follows: *p* < 0.05 (*), *p* < 0.01 (**), *p* < 0.001 (***), *p* < 0.0001 (****).

Plants exposed to the indirect application of biocontrol strains showed that PAL activity in uninfected plants was significantly higher in treatments with strains MRh275 and JRh226 compared with the uninfected control (Figure 4A). After pathogen infection, only strain JRh266 induced a significant increase relative to the infected control (Figure 4B). For the direct biocontrol approach, PAL activity in uninfected plants did not differ significantly among treatments (Figure 4C). In contrast, when the pathogen and biocontrol strains were applied simultaneously, strains MRh275 and JRh266 showed reduced PAL activity, whereas strain JRh226 induced significantly higher activity compared with the infected control (Figure 4D).

### 2.6. Expression of Defense-Related Genes in Response to Biocontrol Treatments

**Figure 5:**
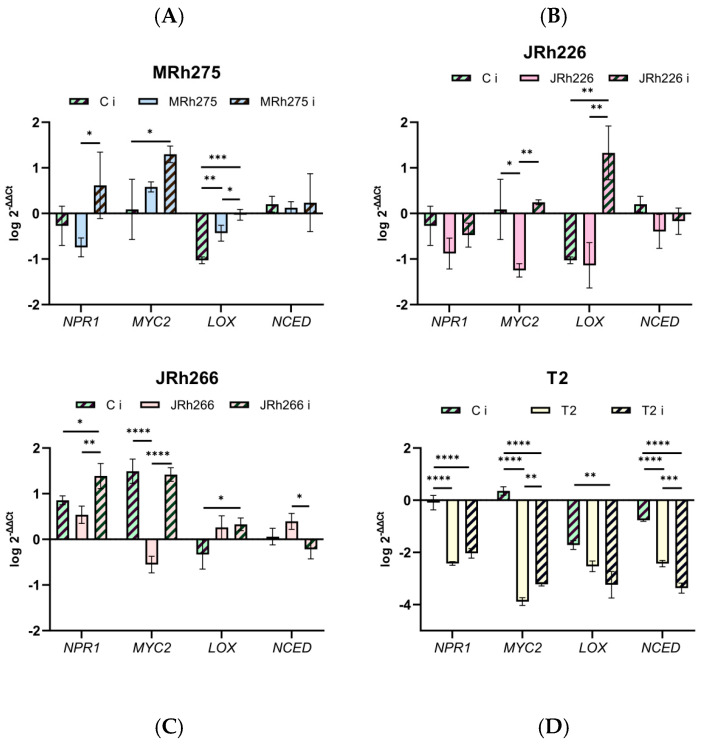
Genes *NPR1*, *MYC2*, *LOX*, and *NCED* expression were measured in sugar beet leaves under indirect biocontrol conditions. Seeds were treated with biocontrol strains: MRh275 (**A**), JRh226 (**B**), JRh266 (**C**) or the T2 consortium (**D**). Gene expression was analyzed in both uninfected plants and plants infected with *P. syringae* pv. *aptata* P21. The infected control group (C i) was not exposed to bacteria before infection. Gene expression in C i was compared with both infected (MRh275 i, JRh226 i, JRh266 i, and T2 i) and uninfected (MRh275, JRh226, JRh266, and T2) biocontrol-treated plants. Comparisons were also made between uninfected and infected pretreated plants. Relative expression levels were normalized to the *25S rRNA* housekeeping gene, using the uninfected untreated control as the reference. Significant differences are indicated as follows: *p* < 0.05 (*), *p* < 0.01 (**), *p* < 0.001 (***), *p* < 0.0001 (****).

The expression of defense-related genes, *NPR1*, *MYC2*, *LOX*, and *NCED*, was analyzed by qPCR to evaluate the effects of biocontrol treatments on plant defense signaling pathways. Under the indirect biocontrol approach, the expression patterns of the analyzed defense-related genes varied depending on the applied biocontrol strain (Figure 5). For strain MRh275 (Figure 5A), no significant differences in *NPR1* expression were detected between the infected control (C i) and plants treated with MRh275, nor between C i and infected plants treated with MRh275, whereas a significant increase was observed between uninfected (MRh275) and infected plants (MRH275 i) treated with MRh275. For *MYC2*, transcript levels were significantly higher only in the treated infected plants. For *LOX*, treatment with the MRh275 strain leading to higher expression compared with C i, while *NCED* expression did not differ significantly among the analyzed treatments.

For strain JRh226 (Figure 5B), *NPR1* and *NCED* expression remained unchanged across treatments. *MYC2* transcript levels were significantly lower in JRh226 compared with the infected control, as well as in infected plants treated with JRh226. For LOX, JRh226 i exhibited significantly higher expression compared with both C i and JRh226.

In plants treated with strain JRh266 (Figure 5C), infected plants exhibited significantly higher *NPR1* expression compared with both JRh266 and the infected control. For *MYC2*, the infected control showed higher transcript levels I in treated plants, reflecting the trend seen for strain JRh226. In addition, *LOX* expression in treated infected plants was significantly higher than in C i. For *NCED*, expression was significantly higher in uninfected plants treated with that strain than in infected treated plants.

In contrast, plants treated with the T2 consortium (Figure 5D) generally exhibited reduced expression of the analyzed genes. The infected control showed significantly higher *NPR1*, *MYC2*, *LOX* and *NCED* expression compared with both T2 and infected plants treated with T2. Notably, all treatments exhibited expression levels lower than those observed in healthy plants.

**Figure 6:**
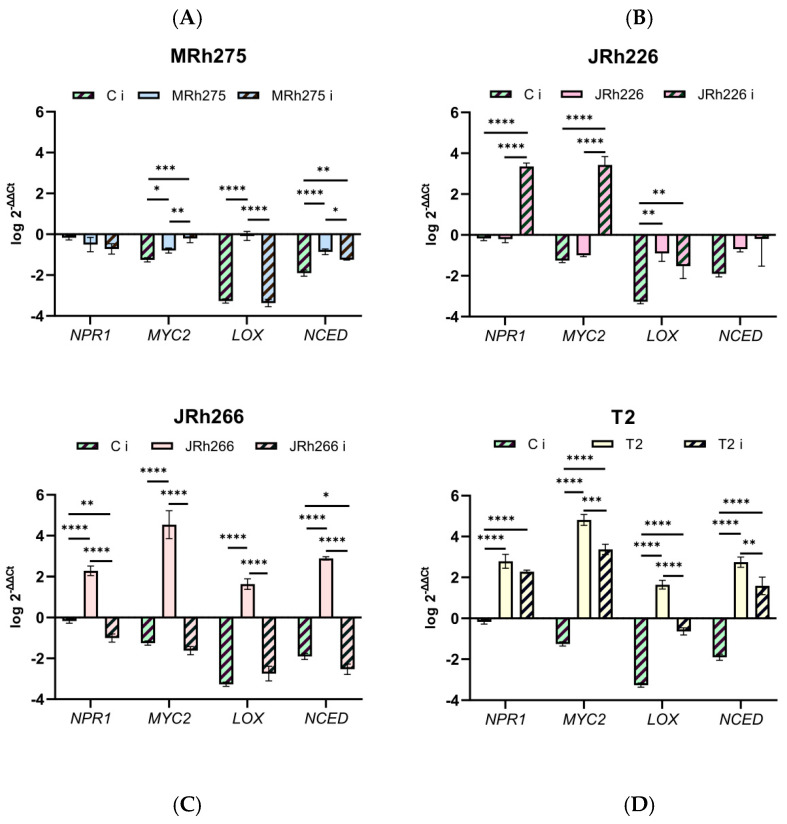
Genes *NPR1*, *MYC2*, *LOX*, and *NCED* expression in sugar beet leaves under direct biocontrol conditions. Plants were inoculated with either a biocontrol strain: MRh275 (**A**), JRh226 (**B**), JRh266 (**C**) or the T2 consortium (**D**) alone, or simultaneously with the biocontrol strain or the T2 consortium and the pathogen *P. syringae* pv. *aptata* P21. The infected control group (C i) was not exposed to bacteria before infection. Gene expression in C i was compared with both infected (MRh275 i, JRh226 i, JRh266 i, and T2 i) and uninfected (MRh275, JRh226, JRh266, and T2) biocontrol-treated plants. Additionally, comparisons were made between uninfected and infected pretreated plants. Relative expression levels were normalized to the *25S rRNA* housekeeping gene, using the uninfected untreated control as the reference. Significant differences are indicated as follows: *p* < 0.05 (*), *p* < 0.01 (**), *p* < 0.001 (***), *p* < 0.0001 (****).

The expression of the same set of defense-related genes under the direct biocontrol approach is shown in Figure 6. For strain MRh275 (Figure 6A), no significant differences in *NPR1* expression were observed among the treatments analyzed. For *MYC2*, transcript levels in the infected control (C i) were significantly lower than in both plants treated with MRh275 and infected plants treated with the biocontrol strain (MRh275 i). For *LOX*, both C i and MRh275 i showed significantly lower expression compared with MRh275. For NCED, both MRh275 and MRh275 i exhibited significantly higher transcript levels than the infected control, with MRh275 showing higher expression than MRh275 i. However, all gene values remained below those observed in healthy plants.

For strain JRh226 (Figure 6B), infected plants treated with the biocontrol strain (JRh226 i) exhibited significantly higher *NPR1* and *MYC2* expression compared with both the infected control (C i) and plants treated with the strain alone (JRh226). For *LOX*, both JRh226 and JRh226 i showed significantly higher expression than C i, although expression levels in all treatments remained lower than in healthy plants. No statistically significant differences were detected for *NCED* expression.

In plants treated with strain JRh266 (Figure 6C), *NPR1* expression was significantly higher in JRh266 compared with both C i and infected plants treated with the strain (JRh266 i), while the infected control showed higher expression than JRh266 i. A similar pattern was observed for *MYC2*, *LOX*, and *NCED*, where JRh266 exhibited significantly higher transcript levels than both C i and JRh266 i, whereas for *NCED*, C i remained higher than JRh266 i.

For consortium T2 (Figure 6D), both T2 and infected plants treated with the consortium (T2 i) showed significantly higher *NPR1* expression compared with the infected control. The same trend was observed for *MYC2*, *LOX,* and *NCED,* although transcript levels in T2 were significantly higher than in T2 i.

## 3. Discussion

In this study, we compared the efficacy and mechanisms of indirect and direct biocontrol strategies using individual bacterial strains and the T2 consortium against *Pseudomonas syringae* pv. *aptata* P21 in sugar beet. Direct co-inoculation of biocontrol agents with the pathogen resulted in a clear reduction in lesion development, indicating immediate antagonistic interactions. Based on our previous in vitro results [17], this was our expectation. However, our results show that indirect biocontrol produces more pronounced and consistent effects on plant defense activation. Under indirect treatment, higher H_2_O_2_ levels in uninfected plants treated with individual strains indicate controlled activation of oxidative signaling. Similar findings have been reported for pepper plants treated with *Pseudomonas putida* A32 [18], which showed significantly increased H_2_O_2_ levels under uninfected conditions, reflecting signaling events associated with microbial colonization [19]. In contrast, the T2 consortium maintained lower basal levels, leaving plants susceptible to infection. This pattern suggests more balanced redox regulation that enables signaling without excessive oxidative damage [20]. Following pathogen infection, H_2_O_2_ concentration increased significantly only in plants treated with strain *Bacillus pseudomycoides* JRh226, whereas other treatments showed reduced levels compared to the infected control. This suggests that, while strain JRh226 may prime oxidative signaling, the other treatments promote controlled redox regulation, limiting excessive ROS accumulation and oxidative stress [21]. In contrast, direct treatments showed more variable H_2_O_2_ concentrations. In some cases, increased H_2_O_2_ accumulation under infection may reflect stress responses induced by the simultaneous presence of the pathogen. These differences highlight the importance of redox homeostasis in distinguishing regulated defense activation from stress-induced responses [22,23]. Together, these patterns indicate that H_2_O_2_ concentration reflects the balance between signaling and stress, depending on the mode of biocontrol application.

SOD serves as the first line of defense in all aerobic organisms. Its activity generates H_2_O_2_, which is subsequently removed by other enzymes, including POD. In our results, the indirect biocontrol approach increased SOD activity even in uninfected plants treated with *Stenotrophomonas maltophilia* JRh266 and the T2 consortium, while post-infection responses were more pronounced for both SOD and POD, suggesting treatment-specific modulation of defense pathways. Similar patterns of enhanced antioxidant enzyme activities, especially under pathogen infection, have been reported in peanut plants treated with the rhizobacterium *Bacillus siamensis* YB-1632 [24]. The observed increase in POD activity after infection in both indirect and direct approaches reflects peroxidase’s role in plant physiology, including H_2_O_2_ scavenging, lignification, wound healing, and regulation of cell elongation [25]. Additionally, increased POD activity after infection may contribute not only to reactive oxygen species metabolism but also to cell wall reinforcement through oxidative cross-linking of structural polymers, potentially enhancing resistance to subsequent pathogen invasion [21,26]. Elevated antioxidant enzyme activity upon pathogen infection is crucial for preserving cell membrane integrity, regulating intracellular osmotic pressure, mitigating membrane lipid peroxidation, and reducing the generation of reactive oxygen species (ROS) under drought stress conditions [21]. In contrast, direct biocontrol treatments resulted in a specific and consistent pattern of antioxidant enzyme modulation characterized by increased POD activity and decreased SOD activity in uninfected plants. These changes likely reflect regulation of ROS signaling and basal plant defenses, without evidence of basic priming [26]. Following pathogen infection, POD activity remained significantly elevated in certain treatments, while SOD showed no statistically significant changes, indicating a primary role for POD in the induced defense response. Similar patterns of antioxidant enzyme modulation have also been reported in other plant systems [27]. These responses are consistent with induced systemic resistance, where beneficial microbes enhance the plant’s capacity to respond to pathogen infection without necessarily triggering constitutive defense activation [8,28,29].

The priming capacity of indirect biocontrol is further supported by results for phenylalanine ammonia-lyase (PAL) activity. Increased PAL activity in uninfected plants treated with *B. safensis* MRh275 and *B. pseudomycoides* JRh226 indicates pre-activation of the phenylpropanoid pathway, suggesting an early metabolic shift toward defense-related biosynthesis. This shift likely redirects carbon flux toward secondary metabolism, enhancing the plant’s ability to rapidly deploy structural and antimicrobial defenses upon pathogen infection. The association of PAL activity with redox regulation also highlights its role in coordinating metabolic and signaling components of the defense response. PAL activity is linked to redox balance and coordination with other defense pathways, supporting enhanced readiness for subsequent pathogen infection [30]. In infected plants, PAL activity was elevated in those treated with strain *S. maltophilia* JRh266, reflecting a reactive, infection-induced activation of the phenylpropanoid pathway. This response is consistent with PAL-driven metabolic redirection, in which phytoalexin production limits pathogen growth and lignin deposition at infection sites reinforces structural barriers that confine invasion [31]. In contrast, direct biocontrol treatments did not induce PAL activity in uninfected plants, indicating that this metabolic defense pathway is not pre-activated. Following pathogen infection, PAL activity increased significantly only in plants treated with strain *B. pseudomycoides* JRh226, indicating a possible mechanism involved in the significant reduction in lesions when this biocontrol strain is used. These findings suggest that, unlike indirect biocontrol, which may strengthen basal immunity, direct approaches exhibit a more variable, strain-dependent induction of metabolic defenses [7,32].

Under indirect biocontrol conditions, the T2 consortium did not consistently outperform the most effective individual strains in reducing lesion size. However, it often induced stronger and more balanced biochemical responses. Notably, uninfected plants treated with the T2 consortium showed higher SOD and POD activities and lower H_2_O_2_ levels, suggesting enhanced priming capacity and a more tightly regulated oxidative balance. These effects were most pronounced under indirect application in the absence of infection, indicating that the primary advantage of the T2 consortium lies in preparing the plant for a faster and more controlled stress response rather than direct pathogen suppression, which aligns with current understanding of microbe-induced priming and plant-microbiome interactions [12,33,34]. In contrast, under direct application, a different pattern emerged. The T2 consortium achieved the greatest reduction in lesion size, outperforming individual strains. In these conditions, the consortium reduced H_2_O_2_ accumulation and induced higher enzyme activities, although not significantly, as well as higher gene expression. Similar findings have been reported in a study by Berendsen et al. [35] showing that disease conditions can promote the assembly of beneficial microbial consortia with enhanced protective effects compared to individual strains, highlighting the importance of synergistic interactions in plant defense. Such effects likely result from the combination of complementary traits, including metabolite production, improved niche colonization, and the simultaneous activation of multiple defense pathways, as reported for beneficial microbial consortia [36,37]. Taken together, these findings suggest that combining strains does not necessarily lead to universally additive effects [38], but rather to context-dependent interactions shaped by application method and plant physiological state. Microbial consortia can expand functional potential while also introducing complex inter-strain dynamics, where the balance between cooperation and competition ultimately determines overall performance [39,40].

To clarify the molecular basis of both biocontrol approaches investigated in this study and their relationship with mechanisms involved in induced systemic resistance (ISR) in sugar beet, we analyzed the transcriptional profiles of four key genes representing canonical defense and stress signaling pathways: *NPR1 (NONEXPRESSOR OF PATHOGENESIS-RELATED GENES 1)*, *MYC2 (MYELOCYTOMATOSIS 2)*, *LOX (LIPOXYGENASE)*, and *NCED (NINE-CIS-EPOXYCAROTENOID DIOXYGENASE)* [41]. *NPR1* is the central regulator of the salicylic acid (SA) signaling cascade and is essential for establishing systemic resistance. *MYC2* functions as a major transcription factor in the jasmonic acid (JA) pathway, coordinating defense gene activation. *LOX* encodes lipoxygenase, a crucial enzyme in JA biosynthesis that contributes to both local and systemic immune responses. *NCED* encodes the rate-limiting enzyme in abscisic acid (ABA) biosynthesis, mediating stomatal closure and enhancing resistance against stomata-invading pathogens such as *Pseudomonas syringae*. Integrating gene expression data with H_2_O_2_ concentration, antioxidant, and PAL enzyme activities reveals coordinated defense modulation under both indirect and direct biocontrol strategies. In indirect biocontrol, upregulation of *LOX* in plants treated with strains *B. safensis* MRh275 and *S. maltophilia* JRh266, along with increased *MYC2* and *NPR1* expression primarily during infection, indicates priming of jasmonic acid and salicylic acid signaling pathways [41,42]. These results, together with increased SOD and POD activities, especially after infection, and tightly regulated H_2_O_2_ concentration, which is slightly reduced in infected plants, indicate controlled oxidative signaling rather than stress-induced accumulation [21,43]. A similar pattern of coordinated antioxidant enzyme activation and controlled H_2_O_2_ dynamics under priming conditions has also been reported by Nedeljković et al. [44], further supporting the role of regulated redox signaling in plant defense. Increased PAL activity after *S. maltophilia* JRh266 seed biopriming contributes to enhanced structural and chemical defenses, including lignin deposition and phytoalexin accumulation, supporting more effective resistance against pathogen invasion. Together, these responses indicate that indirect biocontrol establishes a balanced and responsive defense network, where defense genes, oxidative enzymes, and signaling molecules are potentiated without imposing metabolic stress, ultimately contributing to reduced lesion development and enhanced resilience [8,33,45].

In contrast, direct biocontrol generally induced stronger but less coordinated responses. Genes related to three different pathways—SA, JA, and ABA—were significantly upregulated in plants treated with strain *B. pseudomycoides* JRh226 and the T2 consortium in infected and uninfected plants, respectively. Additionally, in T2-treated plants, gene expression was often higher in the biocontrol-only treatment than in the combined pathogen and biocontrol treatment, suggesting that the presence of the pathogen may attenuate biocontrol-induced signaling [46,47]. Enzymatic activity reflected the transcriptional pattern, while H_2_O_2_ levels generally decreased in infected plants treated with strain *B. pseudomycoides* JRh226 and the T2 consortium, indicating a reactive, stress-associated defense mode rather than coordinated redox priming. These findings highlight that the efficacy of biocontrol is not determined solely by pathogen suppression. The plant’s ability to coordinate transcriptional and biochemical defenses, particularly under indirect strategies, provides a more sustainable and energetically efficient enhancement of immunity [28,48,49,50,51].

## 4. Materials and Methods

### 4.1. Selection of Biocontrol Strains

The biocontrol bacterial strains used in this study were *Bacillus safensis* MRh275, *B. pseudomycoides* JRh226, and *Stenotrophomonas maltophilia* JRh266, isolated from the rhizosphere of sugar beet, as previously described [17]. These strains were selected from 98 strains for their antibacterial activity against different pathogenic *P. syringae* strains and fungi, and for their plant growth-promoting (PGP) traits, presented in the same study. All 98 strains were further characterized for the following traits. Indole-3-acetic acid (IAA) production was detected using the method described by Mohite [52]. Bacterial isolates were grown in Luria–Bertani broth (LB) (all components from Torlak, Belgrade, Serbia) supplemented with tryptophan at 30 °C with shaking (IKA-Werke GmbH & Co. KG, Staufen, Germany) at 180 rpm for 7 days, and IAA production was quantified using the Salkowski reagent by measuring absorbance at 535 nm. The ability of the isolates to fix nitrogen was assessed on nitrogen-free bromothymol blue (NFB) medium prepared according to Baldani et al. [53], where the formation of a white pellicle indicated nitrogen-fixing capacity. ACC deaminase activity was determined following the method of Dworkin and Foster [54] and quantified spectrophotometrically (Thermo Scientific, Waltham, MA, USA) at 540 nm based on the production of α-ketobutyrate from ACC using a standard curve. Swimming, swarming, and twitching motility were evaluated on semi-solid media according to Palma et al. [55], and motility was assessed by measuring the diameter of bacterial migration after 24–48 h of incubation at 30 °C. Biofilm formation was determined using the microtiter plate assay described by Stepanović et al. [56]. Briefly, the suspension of each isolate in tryptic soy broth (TSB) (TM Media, Titan Biotech LTD, Rajasthan, India) was adjusted to a density of 0.5 McFarland (Biosan, Riga, Latvia). The cultures were then diluted 1:100 in 200 μL TSB and inoculated into the wells of a flat-bottomed polystyrene 96-well plate (Sarstedt AG & Co. KG, Nümbrecht Germany). *Pseudomonas aeruginosa* PAO1 was used as the positive control, and sterile TSB medium served as the negative control. The plates were incubated at 37 °C for 24 h, and wells were subsequently washed three times with sterile PBS (pH 7.2). Adherent biofilms were fixed for 30 min at 65 °C, stained for 30 min at room temperature with 200 μL of 0.1% crystal violet, then rinsed in still water and dried at 65 °C. Biofilms were resolubilized with 200 μL of a solution containing 96% ethanol and acetone in a 4:1 ratio for 15 min, and the OD was read at 595 nm. The low cut-off (ODc) was calculated as three standard deviations above the mean OD of control wells. Strains were classified according to the following criteria: no biofilm producer (OD ≤ ODc), weak biofilm producer (ODc < OD ≤ 2 × ODc), moderate biofilm producer (2 × ODc < OD ≤ 4 × ODc), and strong biofilm producer (4 × ODc < OD).

The plant pathogen used for the infection assay was *P. syringae* pv. *aptata* P21, which was also isolated from sugar beet [57]. All bacterial strains were cultured on Luria–Bertani agar (LA) plates at 30 °C. For inoculum preparation, single colonies were transferred into LB and incubated at 30 °C for 24 h with constant agitation at 180 rpm.

### 4.2. Consortium Formulation

Strains *B. safensis* MRh275, *B. pseudomycoides* JRh226, and *S. maltophilia* JRh266 were tested in a cross-antagonistic assay using the spot on the lawn method and the well diffusion assay. Each strain served as both the sensitive and the antagonistic strain. In the first test, 10 µL of a 16 h overnight culture was dropped onto the surface of soft LA (0.7% agar) in which the sensitive strain had previously been inoculated at the 10^5^ CFU mL^−1^ final concentration. In the second test, wells were made in the soft LA with sensitive strain, and 50 µL of the 16 h overnight culture was added to each well. The Petri dishes were incubated overnight at 30 °C.

Growth curves for the individual strains were first established in LB medium by monitoring viable cell counts over time. The optical density at 600 nm (OD_600_) of overnight cultures was correlated with colony-forming units (CFU mL^−1^) to generate calibration curves for estimating bacterial concentrations. For co-culture experiments, overnight cultures were adjusted to achieve equal initial cell densities for each strain and combined to form the consortium. The mixed culture was inoculated into fresh LB medium and incubated at 30 °C with shaking at 180 rpm. At defined time intervals, samples were serially diluted and plated on Luria–Bertani agar (LA) to determine CFU counts. Individual strains within the consortium were distinguished by their distinct colony morphology on LA plates. Growth curves for each strain in the consortium were constructed based on viable cell counts.

Strains *B. safensis* MRh275 and *S. maltophilia* JRh266 selected for consortium formulation were grown overnight, OD600 was measured, the culture was centrifuged, washed with sterile water, and adjusted to 10^9^ CFU mL^−1^ for indirect and 10^8^ CFU mL^−1^ direct biocontrol treatment. The bacterial consortium was prepared by mixing equal volumes of the individual bacterial suspensions of MRh275 and JRh266 (1:1, *v*/*v*). The combined suspension was homogenized under sterile conditions and prepared immediately before application.

### 4.3. Indirect and Direct Biocontrol Approach and Infection Assay

Two biocontrol experimental approaches were used *in planta*. In the indirect biocontrol method, surface-sterilized seeds [17] were treated with bacterial suspensions (10^9^ CFU mL^−1^) of the selected biocontrol strain or consortium for 1 h before sowing. Control seeds were sterilized and treated with sterile distilled water. All seeds were sown in plastic pots (10 × 10 × 10 cm) containing sterile commercial substrate (Plagron LightMix, Ospel, The Netherlands). The pots were maintained in a growth box under controlled conditions (20 ± 2 °C, 16/8 h light/dark photoperiod) and watered regularly. The experiment followed a completely randomized design, and pot positions were rotated weekly to minimize microclimatic variation. After germination and growth to the four-leaf stage, plants within each treatment were divided into two groups: one group was inoculated with *P. syringae* pv. *aptata* P21 (10^4^ CFU mL^−1^), while the other group remained non-inoculated. Two control groups were included: untreated non-inoculated plants (C) and untreated pathogen-inoculated plants (Ci). In the direct biocontrol approach, all seeds were surface-sterilized and sown without prior bacterial treatment. At the four-leaf stage, plants were assigned to four groups: non-inoculated control plants (C), plants inoculated only with the pathogen (10^4^ CFU mL^−1^) (Ci), plants inoculated only with the biocontrol strain (10^8^ CFU mL^−1^), and plants co-inoculated with the mixture of biocontrol strain (10^8^ CFU mL^−1^) and the pathogen (10^4^ CFU mL^−1^) at a 1:4 ratio.

In all treatments, 100 µL of the corresponding suspension was applied into the abaxial side of three fully developed leaves on one plant using a sterile syringe. Leaves were collected five days after inoculation and scanned with a computer scanner. The necrotic lesion area was measured using ImageJ (Fiji bundle, v2.9.0, Bethesda, MD, USA). Afterwards, leaf material was ground in liquid nitrogen and stored at −80 °C for further analysis.

### 4.4. Enzyme Extracts Preparation and Protein Quantification

Two enzyme extracts were prepared from liquid nitrogen–ground leaf tissue. Enzyme extract 1 (EE1) was prepared as described by Lozo et al. [58] using 50 mM potassium phosphate buffer (pH 7) containing 1 mM EDTA, then centrifuged at 13,000 rpm for 30 min at 4 °C. Enzyme extract 2 (EE2) was prepared with 50 mM Tris-HCl buffer (pH 7.5) and centrifuged at 18,000 rpm for 20 min at 4 °C. Supernatants were collected and stored at −80 °C. Protein concentration was measured using the Bradford assay [59] (Thermo Scientific, Waltham, MA, USA) by mixing 25 µL of extract with 750 µL of reagent, incubating for 5 min at room temperature, and measuring absorbance at 595 nm. Concentrations were determined from a standard curve.

### 4.5. Determination of Leaves’ H_2_O_2_ Level

H_2_O_2_ content in sugar beet leaves was measured spectrophotometrically using the KI oxidation method [60]. Briefly, 150 mg of leaf tissue, ground in liquid nitrogen, was homogenized on ice in 1 mL of extraction buffer (0.25 mL 0.1% TCA, 0.5 mL 1 M KI, 0.25 mL 10 mM potassium phosphate, pH 5.8) in the dark. Homogenates were centrifuged twice at 13,000 rpm for 10 min at 4 °C, and the supernatant was incubated in the dark for 20 min. Absorbance was measured at 350 nm, and H_2_O_2_ content was calculated using the molar extinction coefficient (nmol g^−1^ fresh tissue).

### 4.6. Antioxidant Enzyme Activity Determination

The activities of the antioxidant enzymes total soluble peroxidases (POD) and superoxide dismutase (SOD) were measured spectrophotometrically using previously prepared protein extracts, following the method described by Lozo et al. [58]. EE1 was used for SOD measurements, and EE2 was used for POD activity. SOD activity was evaluated at 560 nm by monitoring inhibition of the photochemical reduction of nitro blue tetrazolium. POD activity was measured at 470 nm based on guaiacol oxidation.

### 4.7. Phenylalanine Ammonia-Lyase (PAL) Activity

PAL activity was measured spectrophotometrically by monitoring the formation of trans-cinnamic acid from L-phenylalanine, following a modified method from Kleinhofs et al. [61]. The assay involved mixing 100 µL of EE2 with 0.5 mL of 50 mM Tris–HCl buffer (pH 8.8) and 0.6 mL of 1 mM L-phenylalanine. After mixing, samples were incubated at 40 °C for 60 min, and the reaction was stopped by adding 1 mL of 2 N HCl. Absorbance was measured at 290 nm, and enzyme activity was expressed as micrograms of trans-cinnamic acid per gram of leaf tissue.

### 4.8. RNA Extraction, cDNA Synthesis, and Gene Expression Analysis

Leaf tissue was ground in liquid nitrogen, and total RNA was extracted from 70 mg of tissue using the RNeasy^®^ Plant Mini Kit (QIAGEN, Hilden, Germany). RNA quality, integrity, and concentration were assessed with a NanoPhotometer and confirmed by 1% agarose gel electrophoresis. RNA samples were treated with DNase I (RNase-free, Thermo Scientific, Waltham, MA, USA) to remove genomic DNA contamination, and first-strand cDNA was synthesized using the RevertAid First Strand cDNA Synthesis Kit (Thermo Scientific, MA, USA). The quality of the cDNA was verified by agarose gel electrophoresis.

Relative expression of *NPR1 (NONEXPRESSOR OF PATHOGENESIS-RELATED GENES 1)*, *MYC2 (MYELOCYTOMATOSIS 2)*, *LOX (LIPOXYGENASE)*, and *NCED (NINE-CIS-EPOXYCAROTENOID DIOXYGENASE)* genes was determined by quantitative PCR (qPCR) using gene-specific primers and SYBR Green Master Mix on a StepOnePlusTM Real-Time PCR System (Applied Biosystems, Waltham, MA, USA). The primer sequences used for amplification were as follows: *NPR1* (NPR1BvF: 5′-TCATGAAGCTTGTCGTCCTG-3′; NPR1BvR: 5′-ATACACCTTGCCAGCAATCC-3′) [62], *MYC2* (BvMYC2-F: 5′-AGTGAGCTTGACGTGCAGTA-3′; BvMYC2-R: 5′-CCCCTCATCTGCCTCAAGAAATAC-3′), *LOX* (BvLOX-F: 5′-ATCGGCAGTTGAGTGCAATG-3′; BvLOX-R: 5′-CTGCCATTCCCCTGCTTACA-3′) and *NCED* (BvNCED-F: 5′-AGGAACAAACTGGGGAGGAAAA-3′; BvNCED-R: 5′-TCCCAATTGATCTCATCGTGC-3′) [63].

qPCR amplification was performed using the following thermal cycling conditions: initial incubation at 50 °C for 2 min, followed by initial denaturation at 95 °C for 10 min, and 40 amplification cycles consisting of denaturation at 95 °C for 15 s and annealing and elongation at 60 °C for 1 min. Melt curve analysis was performed at the end of the amplification protocol to verify product specificity. Relative gene expression was calculated using the ΔΔCt method with *25S rRNA* as the reference gene (25RNK_F: 5′-AGACAAGAAGGGGCAACGAG-3′; 25RNK_R: 5′-CACATTGGACGGGGCTTTTC-3′) [64], and fold changes were expressed relative to the untreated, non-inoculated control.

### 4.9. Statistical Analysis

All experiments were performed with three biological replicates. Statistical analyses were conducted using GraphPad Prism v9.0.0 for Windows (GraphPad Software, San Diego, CA, USA). Data were analyzed by one-way analysis of variance (ANOVA), followed by Dunnett’s multiple comparisons test when treatments were compared with the control group. Comparisons among individual strains forming the consortium and the consortium itself were performed using ANOVA followed by Šídák’s multiple comparisons test. For qPCR analyses, differences among treatment groups were evaluated by ANOVA followed by Tukey’s multiple comparisons test. A significance level of *p* < 0.05 was used for all analyses.

## 5. Conclusions

From a practical perspective, these findings highlight that biocontrol evaluation should extend beyond visible disease suppression. Direct biocontrol enables rapid reduction of disease symptoms through immediate antagonistic interactions, making it highly effective under acute pathogen pressure. In contrast, indirect biocontrol enhances the plant’s intrinsic defense capacity before infection by establishing a primed state, which improves the speed and efficiency of defense activation upon pathogen infection. This mechanism is particularly relevant under field conditions, where pathogen occurrence is unpredictable and long-term resilience is required [14,27,37]. Consistent with this, our results show that indirect application plays a key role in modulating plant responses at biochemical and molecular levels, as evidenced by coordinated changes in enzymatic activities and gene expression associated with enhanced defense readiness. Although these effects may not always result in the strongest immediate disease suppression, they contribute to more stable and durable protection over time. Overall, this study underscores the importance of integrating host-mediated resistance with direct pathogen suppression and supports the use of indirect biocontrol as a critical component of sustainable plant disease management strategies.

## Figures and Tables

**Table 1:** Plant growth promotion characteristics of selected strains.

Characteristics	Strains		
	*B. safensis* MRh275	*B. pseudomycoides* JRh226	*S. maltophilia* JRh266
Amylase	−	−	−
Proteinase	−	−	−
Gelatinase	−	−	−
Mannanase	−	−	−
Cellulase	+	−	−
Quorum Sensing	−	−	−
Quorum Quenching	+	+	−
Germination	−	−	+
Exopolysaccharides	−	−	−
Siderophores	−	−	−
HCN	−	−	−
Phosphate solubilization	+	−	−
*P. syringae* CFBP2437	+	+	+
*P. syringae* P16	+	−	+
*P. syringae* P21	+	+	+
*F. oxysporum*	+	+	+
*R. solani*	+	+	+
*Cercospora* spp.	+	+	+
IAA	+	+	+
Biofilm	−	+	−
Swimming	−	+	−
Swarming	+	+	−
Twitching	−	−	−
ACC deaminase	+	-	+
Nitrogen fixation	−	+	−

+ detected characteristics; − not detected characteristics.

## Data Availability

All the data are available on request from the corresponding author.

## Supplementary Materials

The following supporting information can be downloaded at: https://www.mdpi.com/article/10.3390/ijms27114672/s1.
