# Supplementary material for: Comparative Biocontrol Efficacy and Mechanisms of Indirect and Direct Application Methods Against Leaf Spot Caused by Pseudomonas syringae pv. aptata in Sugar Beet
Source: Int J Mol Sci. 2026 May 22;27(11):4672. doi: 10.3390/ijms27114672 (PMC13257217; doi:10.3390/ijms27114672)
Supplement: Supplementary file 1 [file ijms-27-04672-s001.zip › Table S1.pdf]

Table S1 Cross-antagonistic assay between selected biocontrol strains

	Antagonistic strains		
Sensitive strains	MRh274	JRh226	JRh266
MRh275	—	+	—
JRh226	—	—	—
JRh266	—	+	—
