# Supplementary figures and images for: Comparative Biocontrol Efficacy and Mechanisms of Indirect and Direct Application Methods Against Leaf Spot Caused by Pseudomonas syringae pv. aptata in Sugar Beet
Source: Int J Mol Sci. 2026 May 22;27(11):4672. doi: 10.3390/ijms27114672 (PMC13257217; doi:10.3390/ijms27114672)

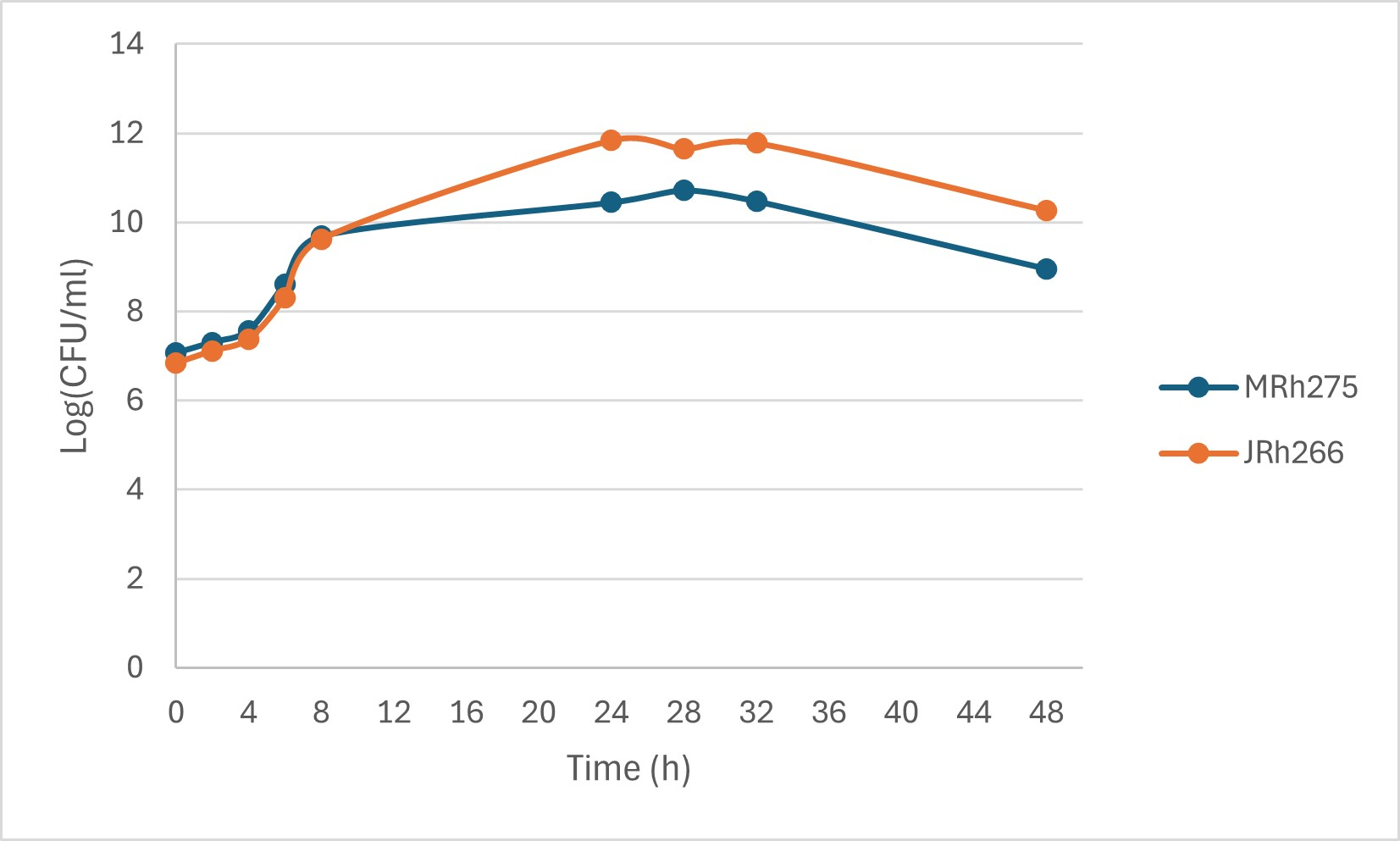

Supplement: Supplementary file 1 [file ijms-27-04672-s001.zip › Figure S1.tif]
